# The impact of CT scan energy on range calculation in proton therapy planning

**DOI:** 10.1120/jacmp.v16i6.5516

**Published:** 2015-11-08

**Authors:** Kevin K. Grantham, Hua Li, Tianyu Zhao, Eric E. Klein

**Affiliations:** ^1^ Nuclear Science and Engineering Institute University of Missouri Columbia MO; ^2^ Department of Radiation Oncology Washington University St. Louis MO USA

**Keywords:** proton therapy, proton planning, CT number, proton stopping power ratio

## Abstract

The purpose of this study was to investigate the impact of tube potential (kVp) on the CT number (HU) to proton stopping power ratio (PSPR) conversion. The range and dosimetric change introduced by a mismatch in kVp used for the CT scan and the HU to PSPR table, based on a specific kVp, used to calculate dose are analyzed. Three HU to PSPR curves, corresponding to three kVp settings on the CT scanner, were created. A treatment plan was created for a single beam in a water phantom passing through a wedge‐shaped bone heterogeneity. The dose was recalculated by changing only the HU to PSPR table used in the dose calculation. The change in the position of the distal 90% isodose line was recorded as a function of heterogeneity thickness along the beam path. The dosimetric impact of a mismatch in kVp between the CT and the HU to PSPR table was investigated by repeating this procedure for five clinical plans comparing DVH data and dose difference distributions. The HU to PSPR tables diverge for CT numbers greater than 200 HU. In the phantom plan, the divergence of the tables resulted in a difference in range of 1.6 mm per cm of bone in the beam path, for the HU used. For the clinical plans, the dosimetric effect of a kVp mismatch depends on the amount of bone in the beam path and the proximity of OARs to the distal range of the planned beams. A mismatch in kVp between the CT and the HU to PSPR table can introduce inaccuracy in the proton beam range. For dense bone, the measured range difference was approximately 1.6 mm per cm of bone along the beam path. However, the clinical cases analyzed showed a range change of 1 mm or less. Caution is merited when such a mismatch may occur.

PACS numbers: 87.55.D, 87.55.Qr

## I. INTRODUCTION

A major source of range uncertainty in proton therapy is in the conversion of computed tomography (CT) number, or Hounsfield units (HU), to the corresponding relative proton stopping power ratios (PSPR) for dose calculation. It is therefore important to characterize the CT simulator used for proton therapy to investigate the possible effects of scanning location, scanning energy, scanning protocol, and the presence of high‐Z material on the measured relationship between the CT number of various materials and their corresponding PSPR.

Of particular interest is the effect of tube potential (kVp) on the conversion. It is well known that the CT number is dependent on the energy spectrum of the scanner.^1^, ^2^ Cropp et al.^(3)^ demonstrated kVp dependence in the CT number. Bai et al.^(4)^ showed a kVp dependence in the determination of the linear attenuation coefficient in PET‐CT and SPECT‐CT applications.

The dependence of CT number on kVp is, in fact, the basis for dual energy CT applications.^1^, ^5^ Based on the aforementioned studies, it should be clear that there would be a kVp dependence for the CT number to PSPR table, as well.

Schneider et al.^(6)^ cited the results presented by McCullough and Holmes,^(7)^ showing that the CT number does not significantly change with scan energy as justification for not including it in their development of the stochiometric method of calculating the CT number to PSPR conversion. Schaffner and Pedroni,^(8)^ following the methods of Schneider and colleagues, did not include the CT scan kVp in their study. Based on these studies, it is common practice for proton therapy clinics to only utilize a single CT number to PSPR table in their treatment planning systems.

The McCullough and Holmes study was limited to scan energies of 120 and 140 kVp. For lower energy scans, such as 80 or 90 kVp, photoelectric interactions would be expected to increase, particularly in high‐Z material such as bone. Increased photoelectric interactions result in an increased measured CT number for high‐Z tissues at low kVp compared to the same tissues at higher energies. Additionally, Mustafa and Jackson^(9)^ showed that the CT number was dependent on the scan kVp, particularly for high‐Z/high‐density materials.

Scanning at a lower kVp might be desirable in order to decrease imaging dose to normal tissue for pediatric patients. Siegel et al.^(10)^ reported a 3.5 cGy reduction in dose for an 80 kVp scan of an 8 cm cylindrical phantom, compared to a 140 kVp scan of the same phantom. The decrease in imaging dose from scanning at a lower kVp is small compared to the therapeutic dose. However, the imaging dose is predominately delivered to normal tissue, so the dose reduction may be beneficial.

Moyers et al.^(11)^ also studied the effect of kVp on the CT number to PSPR conversion. However, that study was limited to water, polycarbonate, polymethylmethacrylate (PMMA), and clear polystyrene. These are relatively low‐Z/low‐density materials (near soft tissue‐equivalent). For these low‐Z materials, Moyers et al. found ±2% difference in the CT number between 80 and 140 kVp compared to 120 kVp.

The purpose of this study is to show the dosimetric effect of a mismatch between the kVp of the planning CT and the kVp of the PSPR conversion table used to calculate dose, demonstrating the need to include verifying the scan energy of the planning CT as part of the patient quality assurance (QA) process.

## II. MATERIALS AND METHODS

### A. CT number–PSPR table creation

The CT simulator used in this study was a Brilliance 16‐slice large bore spiral CT scanner (Philips Healthcare, Andover, MA). Routine quality assurance tests are regularly performed on the CT simulator to ensure their performance is consistent with the recommendations of AAPM Task Group Report 66.^(12)^ Quality assurance test relevant to this study are image noise (checked daily) and CT number accuracy (water tested daily, 7 materials checked monthly and 13 materials checked annually).

**Figure 1:**
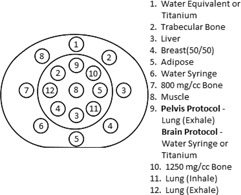
A diagram of the electron density phantom showing the orientation and placement of the materials placed in the phantom for scans using the pelvis scanning protocol. For scans with high‐Z material, the water‐equivalent insert at the top of the phantom was replaced with a titanium insert. For scans using the brain scanning protocol, only the center, circular section of the phantom was scanned with one of the lung (exhale) inserts replaced with either a water syringe or titanium for scans with high‐Z material present.

The CT electron density (ED) phantom (CIRS model 062M, Norfolk, VA) was scanned using a pelvis scan protocol, as shown in Fig. 1. The scan parameters for this protocol were: 90 and 120 kVp, 300 effective mAs, collimator set to 16×1.5 mm, 0.5 s rotation time, 0.688 pitch, standard resolution, and standard filter. The phantom was scanned with a water‐equivalent insert at the top of the phantom (position 1), which was subsequently replaced with a titanium insert for a second set of scans. These scans were repeated with the phantom in three locations within the field of view (FOV) of the CT scanner: in the center of the FOV, at 6 cm up and 6 cm right, and at 5 cm left and 4 cm down. Two additional scans were performed at 140 kVp with the phantom centered in the FOV of the CT with and without the titanium insert.

The same procedure was followed using a brain protocol using only the center, circular section of the phantom shown in Fig. 1. The scan parameters for this protocol were: 90 and 120 kVp, 500 effective mAs, collimator set to 16×1.5 mm, 0.5 s rotation time, 0.563 pitch, standard resolution, and standard filter. Like the pelvis protocol scans, the phantom was scanned at three locations within the FOV of the CT. At each location in the FOV two scans were performed, with titanium and with a liquid water syringe placed at position 9 in Fig. 1. The phantom was only scanned in the center of the FOV for a tube potential of 140 kVp.

Multiple scans were acquired in order to ensure, as best as possible, that the differences in the calculated conversion tables were only due to the kVp setting. A total of 28 scans of the CIRS phantom were acquired, including 12 scans for both 90 and 120 kVp, and an additional 4 scans at 140 kVp. Fewer scan were acquired at 140 kVp because data from the 90 and 120 kVp scans showed no dependence on the position of the phantom within the field of view of the CT scanner.

The CT scans that were acquired were then imported into our commercial treatment planning system (TPS), Eclipse v11 (Varian Oncology Systems, Palo Alto, CA). CT numbers of the tissue substitute materials were measured, using the area profile tool in Eclipse, by recording the mean CT number within the area of interest for each material in the electron density phantom. The measured CT numbers were then sorted by the tube potential at which they were acquired and a mean CT number was calculated for each material averaged over all the phantom setups scanned. Three CT number‐PSPR tables were produced from the mean CT number data, using the stochiometric method described by Schneider et al.^(6)^

### B. Uncertainty in the CT number–PSPR conversion tables

The number of measurements contributing to the average CT number of the tissue substitute material used in the stochiometric calculation varied between 4 and 18, depending on kVp and tissue substitute. For instance, the CT number both inhale and exhale lung‐equivalent material was measured four times for the 140 kVp table. Whereas, the CT numbers of all soft tissue‐equivalent materials were measured 18 times for the 120 kVp tables.

For each kVp and tissue‐equivalent material, the mean and standard deviation (SD) of the measured CT number were calculated. The uncertainty in the calculated CT number‐PSPR tables was calculated by performing the stochiometric calculation using the mean CT number ± SD. The limitations of the stochiometric method of calculating the CT number to PSPR table and uncertainty in the ICRU 44^(13)^ human tissues were not included in this study.

For the 140 kVp table, the CIRS phantom was only scanned at the center of the field of view of the CT simulator. Uncertainty due to phantom position was not explored for this kVp.

The 120 kVp CT number–PSPR conversion table was validated by IROC (Imaging and Radiation Oncology Core, Houston, TX). The tissue substitute materials in the CIRS phantom were used to evaluate the accuracy of the CT number‐PSPR conversion tables with other kVp settings. For each material scanned, the CT number was used to calculate the expected PSPR from the conversion table with the corresponding kVp. The expected PSPR, calculated for a given material, should remain constant provided the kVp used to scan the material matched the kVp of the conversion table.

### C. Phantom verification of range change

Three identical copies of a virtual rectangular water phantom (CT number set to 0 HU) with a wedge‐shaped bone heterogeneity (CT number defined to be 1488 HU, the default value for bone in the TPS) placed in the beam path were created in our TPS. Each copy of the phantom was assigned a different CT number–PSPR table to calculate dose. The PSPR tables assigned to the phantoms corresponded to energies available: 90, 120, and 140 kVp. To measure the effect of scan energy on the proton range calculation, a standard plan was created for each PSPR table and subsequently recalculated with a different PSPR tables via the creation of verification plans.

The difference in range between different PSRP tables was measured by the change in the position of the distal 90% isodose value between standard and verification plans relative to a rectangular structure placed distal to the target. A relationship between bone thickness in the beam path and the difference in proton range was obtained by measuring the change in range for several thicknesses of bone placed in the beam path.

### D. Dosimetric impact of PSPR table kVp mismatch in patients

To demonstrate the dosimetric impact of a mismatch in kVp between the planning CT and the HU to PSPR table, five clinical plans representing three anatomical sites were chosen. A description of the clinical cases used in this study is shown in Table 1, including the prescribed relative biological effective dose in cobalt‐cGy‐equivalent (CcGE). For this study, each clinical plan was treated as if it had been inappropriately scanned using 90 kVp but planned assuming they were scanned at 120 kVp. Therefore, the 120 kVp PSPR table was used to calculate dose. The mismatch between the scan kVp and the PSPR kVp would likely manifest as an underestimation of the range of the proton beams, particularly when bone is present in the beam path, compromising the quality of the delivered plan.

Utilizing the same methods as for the phantom discussed earlier, the dosimetric impact of this hypothetical kVp mismatch can be assessed. Using verification plans to ensure that no change in the beam geometry occurred, the dose for all patient plans was recalculated using the 90 kVp PSPR table rather than the 120 kVp table.

Two methods were used to evaluate the impact of changing the PSPR table. First, the dose‐volume histogram (DVH) data from the clinical plan was compared to the DVH data for the recalculated plan. This method demonstrated a change in the dose distribution has occurred for nearby organs at risk (OAR). However, the method lacks the geometry information to fully visualize how the dose distribution changed by calculating the dose with a PSPR table based on a different kVp.

**Table 1:** A description of the clinical plans used in this study

*Patient*	*Plan Description*	*Number of Fields*	*Perscription Dose (CcGE)*
1	Brain Boost	2	1080
2	Brain	3	4500
3	Prostate Boost	2	3420
4	Right Lung	2	4000
5	Left Lung	3	6000

Visualization of the change in the dose distribution was achieved by exporting the RT‐Dose files for the clinical and recalculated plans to MATLAB R2012a (MathWorks, Natick, MA) where dose from the clinical plan was subtracted from the recalculated plan. This difference was then placed into a new RT‐Dose file that was imported back into the TPS, giving a three‐dimensional visualization of magnitude and location of differences between the two plans.

## III. RESULTS

### A. Energy dependence

**Figure 2:**
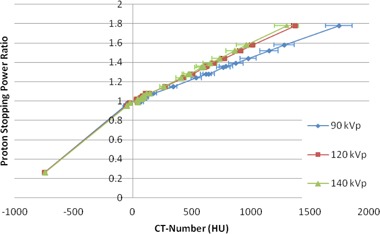
A comparison of the HU–PSPR tables created for two CT simulators and the three available kVps. The error bars represent the uncertainty in the calculated CT number for each of the given ICRU 44^(13)^ tissues used in the stochiometric calculation of the tables.

Figure 2 shows the calculated CT number–PSPR tables for all energies measured. The error bars in Fig. 2 show the uncertainty in the CT number for each human tissue used in the stochiometric calculation of the tables. For comparison between the kVp settings, each table was divided into three sections — low‐density tissue, soft tissue, and high‐density tissue. A linear fit was applied to each section of tissue such that each table was described parametrically by three linear equations. Intercomparison of the tables was then performed by comparing the PSPRs calculated using the linear fit appropriate for the chosen CT numbers.

For tissues with a CT number less than 200 HU, there was less than 3% variation in the calculated PSPR between the different kVp settings. For tissues with a CT number greater than 200 HU, the slopes of the conversion tables decrease with decreasing kVp.

There was an 8% difference in the slope of the linear fits of the 120 kVp and 140 kVp tables for tissues having a CT number greater than 200 HU. This difference in the slope equates to less than 3.5% difference in the calculated PSPR up to a CT number of 2600 HU. For the 90 kVp table, the slope of the linear fit is 23% less than that of the 120 kVp table, resulting in greater than 3.5% difference in the calculated PSPR for tissues with CT numbers above 360 HU. A difference cutoff of 3.5% was chosen because, at that point, the calculated tables are no longer within the accepted uncertainty suggested by Moyers et al.^(14)^

### B. Range shift in phantom

**Figure 3:**
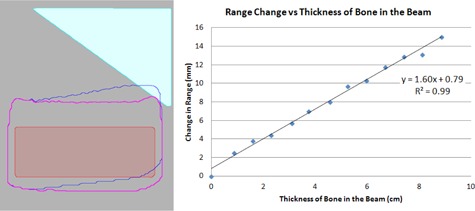
(left) A comparison of the position of the 90% isodose level calculated for the same proton field with the dose calculated using the 90 kVp PSPR conversion table (pink) and the 120 kVp PSPR conversion table (blue). (right) A plot of the change in proton range between the 90 kVp and 120 kVp PSPR tables vs. thickness of bone (CT number=1488) in the beam path. A linear fit to this data gives a range change of 1.6 mm change in range per cm of bone in the beam path. ($R^{2}=0.99$ for the fit).

The calculated range shift seen in the phantom between the 120 kVp and 140 kVp tables was minimal as would be expected given the similarity between the tables. However, when the field calculated using the 90 kVp table was recalculated using the 120 kVp table, the calculated range decreased. Figure 3 shows the change in the 90% isodose level and a plot of the change in the calculated proton beam range versus the thickness of bone in the beam path. The change in range in the phantom was calculated to be 1.6 mm per cm of bone in the beam path for the specific bone material in the phantom.

### C. Patient studies

**Figure 4:**
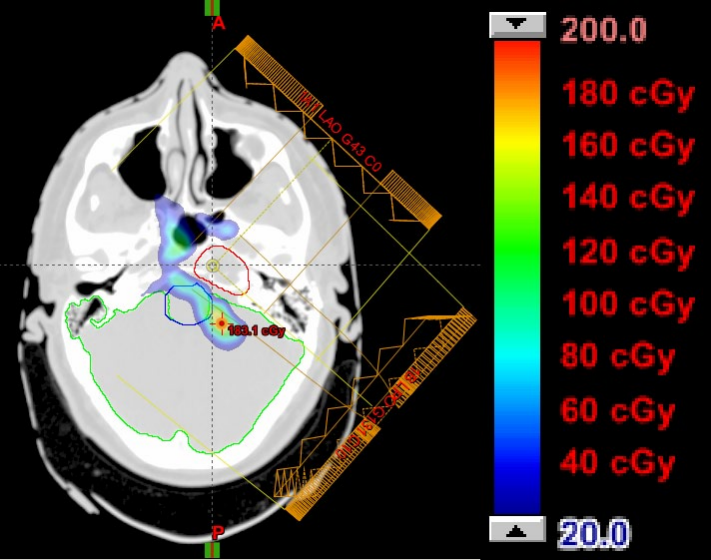
Patient 1, prescribed dose of 1080 CcGE: calculated dose difference for a two‐field boost in the brain. The contours for the CTV, brain, and brainstem are shown.

**Figure 5:**
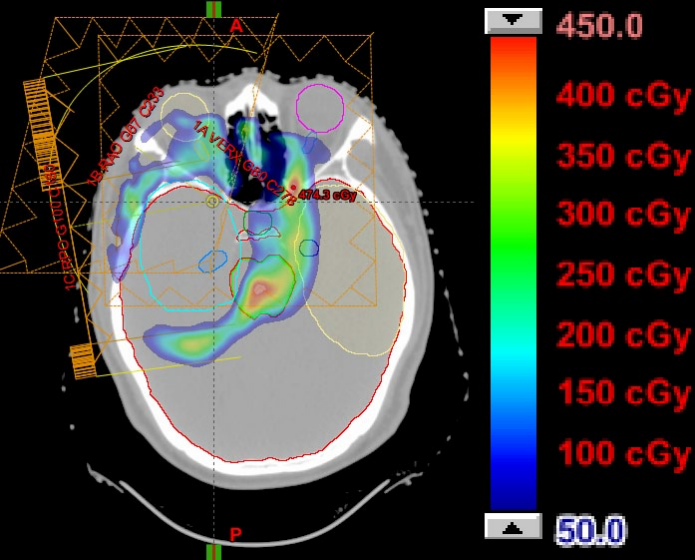
Patient 2, prescribed dose of 4500 CcGE: calculated dose difference for a three‐field plan in the brain. The contours of the CTV, brain, brainstem, left temporal lobe, hippocampus (left and right), pituitary gland, eyes (left and right), and optic nerve (left and right) are shown.

Figures 4 and 5 show the dose difference maps for the two brain treatments. In each figure, the contours for the CTV and relevant critical structures are shown. For Patient 1 (Fig. 4), a mismatch in kVp would result in an increase in the dose to the brain and brainstem. Patient 2 (Fig. 5) also shows an increase in dose to both optic nerves, the pituitary gland, the left temporal lobe of the brain, the left hippocampus, and the brainstem. Additionally, the dose to the right cochlea of Patient 2 would increase by approximately 500 CcGE.

**Figure 6:**
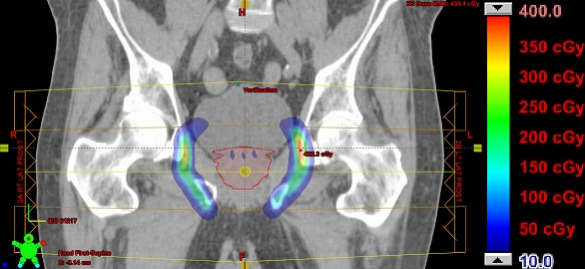
Patient 3, prescribed dose of 3420 CcGE: calculated dose difference for a two‐field boost treatment to the prostate. The contours of the CTV, bladder, and femoral heads are shown.

For the prostate treatment (Patient 3) using opposing lateral beams, there is significant bone in the beam path, but the location of the critical organs is such that the range difference caused by changing the PSPR table is not sufficient to cause a significant change in the DVH data. However, as shown in the dose difference map (Fig. 6), the dose calculated with the 90 kVp table is significantly higher in the normal tissue between the CTV and the femoral heads. This tissue is not associated with a specific OAR. Therefore, it would be unlikely that the dose difference would be detected using DVH analysis.

**Figure 7:**
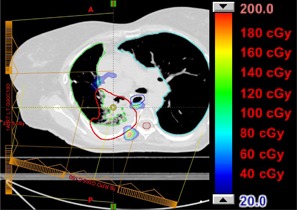
Patient 4, prescribed dose of 4000 CcGE: calculated dose difference for a two‐field treatment to the left lung. The contours of the CTV, lung (left and right), spinal cord, and esophagus are shown.

**Figure 8:**
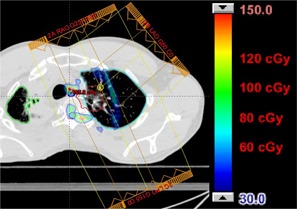
Patient 5, prescribed dose of 6000 CcGE: calculated dose difference for a three‐field treatment to the right lung. The contours of the CTV, lung (left and right), spinal cord, and esophagus are shown.

For the two lung treatments (Patients 4 and 5), the situation is more complex. There were regions with bone present and no critical organ close enough distally to cause a discernible change in the DVH. There are also regions where there is no bone in the proton beam path, and thus no change in range occurs. Finally, there are regions where there is bone present that causes increased dose to a distally located critical organ. However, the volume of the critical organ is sufficiently large compared to the volume of the dose increase that a change in the DVH is not apparent. Figures 7 and 8 show the dose difference maps between the two PSPR tables for the lung treatments. The regions showing the greatest dose difference correlate to regions near the distal edge of the target with the most bone present in the beam path.

## IV. DISCUSSION

A major source of range uncertainty in proton therapy is in the conversion of CT number to the corresponding relative proton stopping power ratios (PSPR) for dose calculation. Schneider et al.^(6)^ and Shaffner and Pedroni^(8)^ showed that using a stochiometric method for calculating the PSPR from the CT number is adequate for obtaining this conversion. However, while beam hardening is included as a source of uncertainty in the CT number, the impact of CT scan kVp on the CT number is not adequately included in their method. Schneider et al.^(6)^ referenced a study by McCullough and Holmes^(7)^ which stated that the CT number does not change significantly with scan energy. We found that, while this is true for soft tissue, for bone the higher Z introduces a higher photoelectric component of attenuation, which would potentially lead to a substantially higher CT number in dense bone for lower energy scans.

In Fig. 2, the error bars represent the uncertainty in the calculated CT number for the given PSPR of the ICRU 44 human tissues modeled. This uncertainty was calculated by performing the stochiometric calculation with the uncertainty in the measured CT number of the tissue‐equivalent materials included.

For the tube potentials used in this study, the uncertainty in the measured CT number of the tissue substitute materials was consistent with the total CT number uncertainties for lung, soft tissue, and bone reported by Yang.^(15)^ In that study, the imaging uncertainty varied from 2.9% at 140 kVp to 3.6% at 80 kVp for lung tissue, 0.58% at 140 kVp to 0.61% at 80 kVp for soft tissue, and 2.1% to 2.5% for bone tissue.

The accuracy of the CT number–PSPR conversion tables was evaluated. For each of the tissue substitute materials used, the PSPR, calculated from the tables, was within 3% of the 120 kVp table for our CT simulator. The 120 kVp conversion table was used as a baseline because it had a third‐party evaluation (IROC).

There are three possible explanations for the difference in the DVH comparisons between the brain and the other cases include: i) either there is not a sufficient amount of bone present in the beam path to cause a significant change in the beam range; ii) the OARs are sufficiently separated from the distal range of the proton beams that the change in the proton range does not change the OAR dose; or, more likely, iii) the explanation for the difference in the DVH comparisons is a combination of both.

When planning for patients with intracranial targets, it is very difficult to place a beam that does not have an OAR near its distal range because everything within the skull is essentially a critical structure. It is also not possible to deliver a beam to the brain that does not pass through bone. The combination of these factors results in the DVH data being different for the same plan calculated using different PSPR tables.

In all the patient cases, the bone in the beam path was much thinner than in the phantom, had a much lower CT number than the bone material in the phantom, or both. Therefore, a mismatch in the kVp of the planning CT and the CT number–PSPR table resulted in a change in the position of the 90% isodose level in the composite dose of 1 mm or less. This suggests that, in many cases, a mismatch in kVp would be unlikely to cause an underdose of the target based on the margins prescribed in the plans. If a patient was scanned at 90 kVp and the dose was calculated using the 120 kVp, the result would be an unaccounted for extension of the range. Any underdose in the target would occur on the proximal side which is more forgiving due to the shallower dose falloff. However, a mismatch could contribute to higher‐than‐expected dose to OARs near the distal edge of a field.

## V. CONCLUSIONS

The degree to which the CT number varies with scan energy creates an increased level of uncertainty in the PSPR for high‐Z material, such as bone, unless the kVp of the planning CT is matched with the kVp of the CT number–PSPR conversion table. This increased uncertainty is minimal for lung and soft tissue.

In the clinical cases studied, the measured change in the position of the 90% isodose level in the composite dose caused by a mismatch in kVp was ∼1 mm. Based on these results, it is likely that current margin definitions (3.5% of the range) may be sufficient to prevent loss of target coverage in many cases. However, a 1 mm increase in the range of a proton field can cause a substantial increase in dose to structures located near the distal edge of the field.

Unlike treatment planning with photons and electrons, a mismatch between the kVp of the planning CT and the kVp of the CT number–PSPR conversion table can substantially impact the quality of the treatment. This study is an important reminder to use caution when a mismatch might occur and to understand what the impact would be for the CT simulator in each proton therapy center.

## References

[1] Brooks RA . A quantitative theory of the Hounsfield unit and its application to dual energy scanning. J Comput Assist Tomogr. 1977;1(4):487–93. 615229 10.1097/00004728-197710000-00016

[2] Yang M , Virshup G , Clayton J , Zhu XR , Mohan R , Dong L . Theoretical variance analysis of single‐ and dual‐energy computed tomography methods for calculating proton stopping power ratios of biological tissues. Phys Med Biol. 2010;55(5):1343–62. 20145291 10.1088/0031-9155/55/5/006

[3] Cropp RJ , Seslija P , Tso D , Thakur Y . Scanner and kVp dependence of measured CT numbers in the ACR CT phantom. J Appl Clin Med Phys. 2013;14(6):338–49. 10.1120/jacmp.v14i6.4417 PMC5714621 24257284

[4] Bai C , Shao L , Da Silva AJ , Zhao Z . A generalized model for the conversion from CT numbers to linear attenuation coefficients. IEEE Trans Nucl Sci. 2003;50(5):1510–15.

[5] Hünemohr N , Krauss B , Tremmel C , Ackermann B , Jäkel O , Greilich S . Experimental verification of ion stopping power prediction from dual energy CT data in tissue surrogates. Phys Med Biol. 2014;59(1):83–96. 24334601 10.1088/0031-9155/59/1/83

[6] Schneider U , Pedroni E , Lomax A . The calibration of CT Hounsfield units for radiotherapy treatment planning. Phys Med Biol. 1996;41(1):111–24. 8685250 10.1088/0031-9155/41/1/009

[7] McCullough EC and Holmes TW . Acceptance testing computerized radiation therapy treatment planning systems: direct utilization of CT scan data. Med Phys. 1985;12(2):237–42. 4000085 10.1118/1.595713

[8] Schaffner B and Pedroni E . The precision of proton range calculations in proton radiotherapy treatment planning: experimental verification of the relation between CT‐HU and proton stopping power. Phys Med Biol. 1998;43(6):1579–92. 9651027 10.1088/0031-9155/43/6/016

[9] Mustafa AA and Jackson DF . The relation between X‐ray CT numbers and charged particle stopping powers and its significance for radiotherapy treatment planning. Phys Med Biol. 1983;28(2):169–76. 6408654 10.1088/0031-9155/28/2/006

[10] Siegel MJ , Schmidt B , Bradley D , Suess C , Hildebolt C . Radiation dose and image quality in pediatric CT: effect of technical factors and phantom size and shape. Radiology. 2004;233(2):515–22. 15358847 10.1148/radiol.2332032107

[11] Moyers MF , Sardesai M , Sun S , Miller DW . Ion stopping powers and CT numbers. Med Dosim. 2010;35(3):179–94. 19931030 10.1016/j.meddos.2009.05.004

[12] Mutic S , Palta JR , Butker EK , et al. Quality assurance for computed‐tomography simulators and the computed tomography‐simulation process: Report of the AAPM Radiation Therapy Committee Task Group No. 66. Med Phys. 2003;30(10):2762–92. 14596315 10.1118/1.1609271

[13] ICRU . Measurements ICRU, Units, ICoR and Measurements. ICRU Report No. 44. Bethesda, MD: International Commission on Radiation Units and Measurements; 1989.

[14] Moyers MF , Miller DW , Bush DA , Slater JD . Methodologies and tools for proton beam design for lung tumors. Int J Radiat Oncol Biol Phys. 2001;49(5):1429–38. 11286851 10.1016/s0360-3016(00)01555-8

[15] Yang M . Dual energy computed tomography for proton therapy treatment planning [GSBS theses]. Houston, TX: University of Texas; 2011.

